# Rod Fracture in Magnetically Controlled Growing Spine Rods

**DOI:** 10.1097/BPO.0000000000002361

**Published:** 2023-02-03

**Authors:** Martina Tognini, Harry Hothi, Sean Bergiers, Edel Broomfield, Stewart Tucker, Johann Henckel, Alister Hart

**Affiliations:** * Royal National Orthopaedic Hospital and Institute of Orthopaedics and Musculoskeletal Science, University College London, Stanmore; † Great Ormond Street Hospital for Children NHS Foundation Trust, London, UK

**Keywords:** MCGR, MAGEC rods, early-onset scoliosis, fracture, growing rods

## Abstract

The mechanisms of fracture in magnetically controlled growing rods (MCGRs) and the risk factors associated with this are poorly understood. This retrospective analysis of explanted MCGRs aimed to add understanding to this subject.

**Methods:**

From our cohort of over 120 retrieved MCGRs, we identified 7 rods that had fractured; all were single-rod constructs, retrieved from 6 patients. These were examined and compared with 15 intact single-rod constructs. Retrieval and fractographic analyses were used to determine the failure mode at the fracture site and the implant's functionality. Cobb angle, degree of rod contouring, and the distance between anchoring points were computed on anteroposterior and lateral radiographs.

**Results:**

5/7 versus 3/15 rods had been inserted after the removal of a previously inserted rod, in the fractured versus control groups. All fractured rods failed due to bending fatigue. Fractured rods had greater rod contouring angles in the frontal plane (*P* = 0.0407) and lateral plane (*P* = 0.0306), and greater distances between anchoring points in both anteroposterior and lateral planes (*P* = 0.0061 and *P* = 0.0074, respectively).

**Conclusions:**

We found all failed due to a fatigue fracture and were virtually all single rod configurations. Fracture initiation points corresponded with mechanical indentation marks induced by the intraoperative rod contouring tool. Fractured rods had undergone greater rod contouring and had greater distances between anchoring points, suggesting that it is preferable to implant double rod constructs in patients with sufficient spinal maturity to avoid this complication.

**Clinical Relevance:**

Level III.

Early-onset scoliosis is defined as a curvature of the spine of more than 10 degrees that is diagnosed before the age of 10 years.^1^ Distraction-based systems are usually used to surgically treat the most severe cases.^2^ The aim of these systems is to control curve progression while allowing for spinal growth until the patient has reached their full development potential.^3^ Traditional growing rods (TGRs) are constructs fixed to the spine using pedicle screws or hooks that are surgically lengthened every 6 to 8 months. Magnetically controlled growing rods (MCGRs), in contrast, do not need repeated rod distraction surgeries performed when a TGR is implanted, thanks to a magnetic distraction mechanism, which allows for outpatient lengthening procedures.^4^

Rod fracture in TGRs is estimated to occur in 15% of the cases^5^; this is one of the most common reasons for the failure of the implant itself.^6^,^7^ A database review of 86 cases of a TGR fracture identified several surgical, implant, and patient risk factors for this, including prior fracture, single rods, small rod diameter, and ambulatory patients.^5^ No correlation between the preoperative severity of the scoliotic curve and rod fracture was found. Several studies investigated the relationship between growing rod fracture and lengthening protocol,^8^,^9^ reaching no consensus. A lower number of studies assessed surgical risk factors (ie, instrumented levels).^10^,^11^ In all cases, it was concluded the failure mode of rod fracture in TGRs was bending fatigue and that stress concentration plays an important role in the rod fracture mechanism.^6^

MCGRs have been shown to experience implant failure in several ways, including an inability to distract due to corrosion or internal pin fracture issues.^12^ A fracture of the MCGR itself is estimated to occur in 10.6% of the total number of complications,^13^ however, the risk factors for this are poorly understood.^14^

The aim of this study was to identify the mechanisms of fracture of explanted fracture MCGRs and assess the risk factors for this by (1) assessing the implant’s state at retrieval (by means of retrieval analysis) (2) analyzing the fracture surface (fractographic analysis), and (3) using imaging analysis to evaluate an eventual patient or surgical risk factors for rod fracture, through comparison with a control group of intact rods.

## METHODS

### Implant and Clinical Data

Of more than 120 retrieved MCGRs sent to our retrieval centre for analysis, this study examined all rods that had fractured. This consisted of 7 rods that had fractured in situ in 6 patients. All implants included in this study were single-rod constructs. One patient in this study experienced consecutive fractures of single rods and both were sent to our centre. One additional fractured double rod construct was retrieved at our centre but it was excluded from the analysis. The rods included in this study were removed by 3 different surgeons across 2 hospitals.

We queried our implant retrieval database to identify 15 intact (ie, not fractured) single-rod constructs with complete clinical data and sufficient imaging to perform a comparison with the cohort of fractured single-rod implants.

Clinical data (age at implantation surgery, sex, time to removal, etiology, and eventual prior surgeries) and implant data (rod configuration, rod size, and rod generation) were recorded for all implants.

**FIGURE 1:**
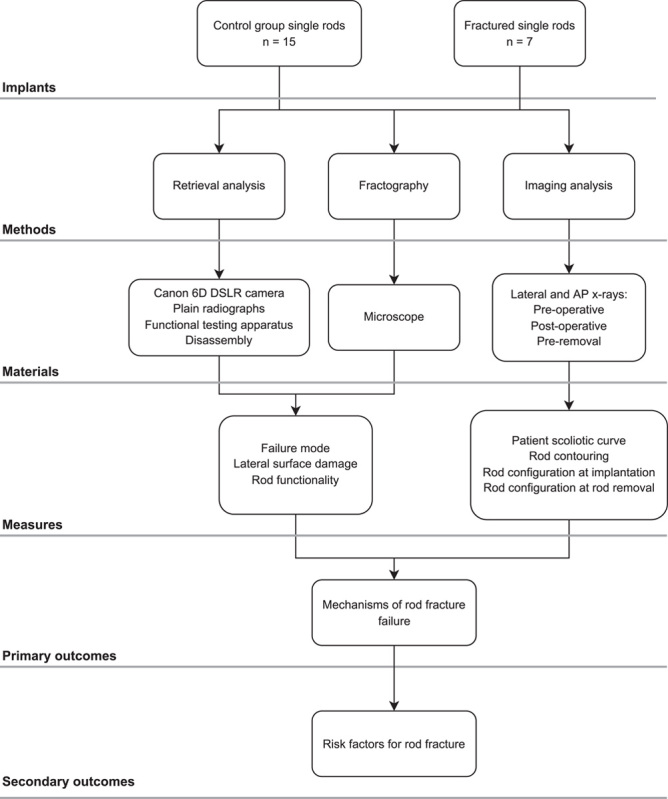
Flowchart showing the study design.

Our study design is summarized in Figure 1.

All implants underwent retrieval, fractographic, and imaging analysis. The primary outcomes assessed in this study are the mechanisms of failure for rod fracture, whereas the secondary outcomes investigated are the risk factors for rod fracture failure.

All patients provided informed consent for their implants and associated clinical data to be investigated at our implant centre. This research was approved by London-Riverside REC: Implant Study—07/Q0401/25.

### Retrieval Analysis

Upon decontamination, all implants underwent retrieval analysis, as described. Visual assessment, plain radiographs, functional testing, and disassembly were used to verify the condition of the implants after removal.

Firstly, a Canon 6D DSLR camera and a Canon 100 mm L lens were used to document the external condition and damage of the implant, together with microscopic analysis (Keyence VHX-700F light microscope, Keyence Co., Japan). The amount of rod distraction reached in vivo was recorded by measuring the distance between the housing tube opening and the first growth mark.

To detect eventual drive pin fracture in the internal mechanism of the implant, plain radiographs were taken using high-energy x-ray scans (Samsung GC85A, Samsung Electronics).

MCGRs showing a functional distraction mechanism (rods able to elongate) underwent elongation testing and uniaxial force testing to measure the maximum achievable elongation and force of the implants after removal. Implants not able to be distracted were sectioned to assess the internal mechanism state.

### Fractography

**FIGURE 2:**
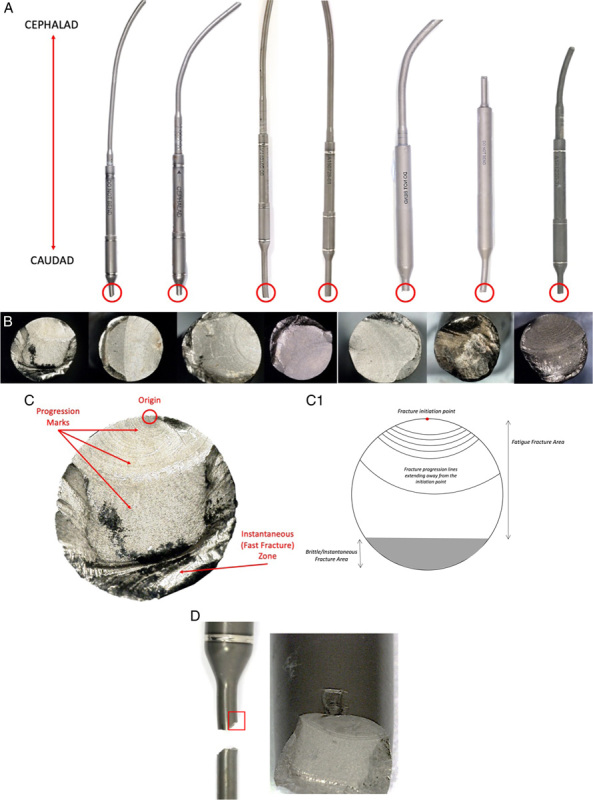
A, Macroscopic picture of fractured rods. Circled in red the area of fracture. All rods fractured on the caudad area of the construct; (B) microscope pictures of the fracture surface of the 7 rods showing clear fatigue failure mode of failure for all constructs. Microscopic image of the fracture surface showing fatigue failure characteristics (C); (D) mechanical indentation in correspondence with the crack initiation point.

Figure 2 summarizes the stages of fractographic analysis performed on the fractured group.

First, the area of fracture was identified on the implant, whether on the telescopic, distraction mechanism or housing tube rod region (Fig. 2A). The fracture surface was then analyzed using a Keyence VHX-700F light microscope (Keyence Co., Japan) to identify the failure mode (ie, fatigue failure), the crack initiation point and the crack propagation direction for each implant, relative to the laser marking on the MCGR (which radially splits the implant into 4 quadrants) (Figs. 2B, C).

Finally, any eventual mechanical indentation on the rod lateral surface in correspondence with the crack initiation point was identified and documented (Fig. 2D). Pictures of the damaged area were taken using a Keyence VHX-700F light microscope (Keyence Co., Japan).

### Imaging Analysis

Preoperative, immediate postoperative, and preremoval anteroposterior (AP) and lateral plain radiographs were retrieved for all patients in this study. All radiographs were calibrated and imaging analysis was performed using Surgimap (Nemaris Inc., New York, NY).

**FIGURE 3:**
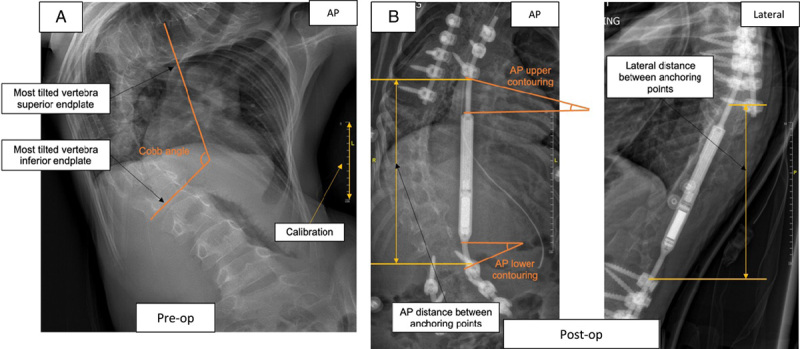
Imaging analysis measurements. A, Preoperative AP radiograph showing the preoperative Cobb angle measurement; (B) postoperative AP and lateral radiograph showing the postoperative measurements, comprising the distance between anchoring points, upper and lower rod contouring degree in the AP and lateral planes. AP indicates anteroposterior.

On preoperative frontal images, the Cobb angle was measured to assess the scoliotic curve severity (Fig. 3A). Postoperative images were used to compare control versus fractured implant configuration at the beginning of treatment. The distance between the most distal upper and most proximal lower anchoring points was computed, both in AP and lateral images. In addition, the rod contouring was quantified by calculating the angle between the housing tube opening and the rod at anchoring (Fig. 3B).

Rod contouring, carried out at implantation surgery to accommodate the implant shape to the spine curvature, differently from other types of growing rods, is possible only outside of the distraction mechanism region. For this reason, an “upper contouring” (on the cephalad region of the implant) and a “lower contouring” (on the caudad region of the implant) were defined and calculated both on AP and lateral radiographs. These measurements were taken to give a preliminary estimation of the mechanical lever the rods were subjected to. In addition, the immediate postoperative Cobb angle was computed.

On immediate preremoval radiographs, the rod fracture area relative to the patient axis was identified. In particular, the location of the fracture relative to the anchoring constructs was noted.

Statistical analysis was performed using GraphPad Prism version 9.0.0 for Mac (GraphPad Software, San Diego, CA). Statistical significance was considered for a *P* value <0.05. The Mann-Whitney *U* test was carried out when comparing numerical data between the two groups, comparing medians across groups.

## RESULTS

### Clinical and Retrieval Data

**FIGURE 4:**
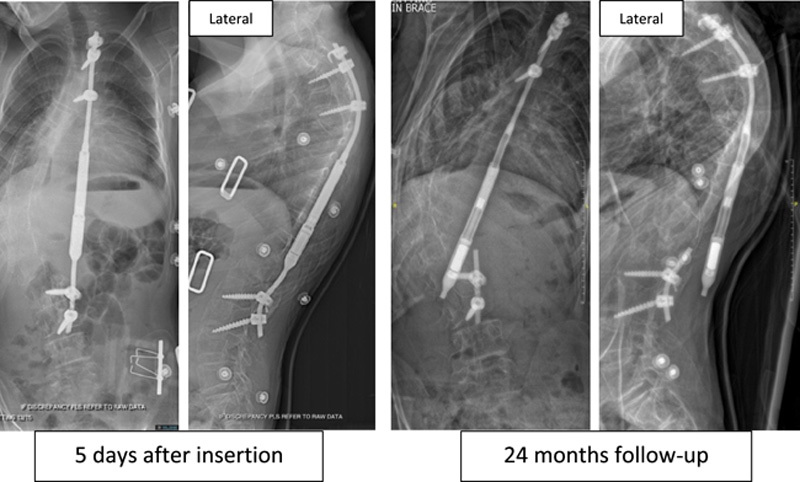
Fractured rod immediate postoperative and at latest follow-up before rod removal. This patient received a single rod after the removal of a precedent fractured rod and was implanted with the second implant for 24 months.

Implant and clinical data results are summarized in Table 1. Age at insertion surgery was higher in the fractured rod group (5 vs 9 years old in the control and fractured groups, respectively) without reaching statistical significance (*P* = 0.0954). Time to removal was significantly lower in the fractured rods group (*P* = 0.0143). Five of the fractured rods had been inserted after the removal of a previously inserted rod (Fig. 4), whereas in the control group, 3 constructs were revisions.

**TABLE 1:** Clinical and Implant Data Results for the Control and the Fractured Implants Groups. The Results are Displayed as Median (Minimum-maximum). Mann-Whitney Nonparametric Test was Used to Compute *P* Values

Data		Groups		
		Control	Fractured	*P*
No. rods evaluated		15	7	—
No. patients		15	6	—
Age at surgery (y)		5 (1-12)	9 (3-13)	0.0954
Time to removal (mo)		29 (14-66)	12 (5-45)	0.0143†
Sex (F)		8	4	—
Etiology			
Idiopathic		4	1	—
Congenital		4	1	—
Neuromuscular		4	3	—
Data not available		3	1	—
Rod exchange*		3	5	—
Length received (mm)		205 (152-325)	238 (141-283)	0.6679
Rod distraction received (mm)		19 (0-40)	10 (0-30)	0.6663
Implant design generation	1.0-1.2	6	1	—
	1.3-1.5	4	4	—
	X	5	2	—
Rod size (length)	70	7	1	—
	90	8	6	—
Rod size, diameter	4.5	8	4	—
	5.0	1	1	—
	5.5	6	2	—
Functional at retrieval		8	5	—

* The rod was not implanted as index surgery.

† Statistically significant *P* values.

Both groups comprised implants from all the design iterations. Rod size is determined by 2 parameters: (1) the actuator region length (70 mm vs 90 mm) and (2) the telescopic rod diameter (4.5 mm, 5 mm, and 5.5 mm). No clear correlation between the size of the implant and the occurrence of rod fracture was noted. Most fractured implants were found functional at retrieval analysis (n = 5/7), versus a lower share of the implants in the control group (n = 8/15).

### Fractography

Rod fracture occurred in all the examined rods at the caudad end of the construct (Fig. 2A). Crack initiation points and fracture propagation waves (typical of fatigue failure mode) were clearly evident in all implants (Fig. 2B). Four implants fractured in correspondence with the anchoring construct, while 3 just above it.

On the lateral surface of the rod, in correspondence with the crack initiation point, mechanical indentation due to mechanical damage during implantation surgery was noted in all MCGRs (Fig. 2D). The same indentations were noted in the control group as well. The mode of failure for all rods seems to be fatigue failure initiating at a mechanical indentation point induced by the bending tool used to contour the construct.

### Imaging Analysis

Imaging analysis results are summarized in Table 2. The severity of the curve (Cobb angle) in preoperative images was similar in the 2 groups (*P* = 0.7574), similar to the postoperative Cobb angle (*P* = 0.6955). The median preoperative Cobb angle in the control group was 81.5 degrees (23 degrees to 120 degrees), whereas in the fractured rods was 65 degrees (33 degrees to 110 degrees). The distance between anchoring points (Fig. 3B) was significantly higher in the fractured rods group, both in the AP and lateral planes (*P* = 0.0061 and *P* = 0.0074, respectively). Rod contouring angles were higher in the fractured implants group (*P* = 0.0407 for the AP upper contouring angle and *P* = 0.0306 for the lateral lower contouring angle).

**TABLE 2:** Imaging Measurements Results. The Results are Displayed as Median (Minimum-maximum). Mann-Whitney Nonparametric Test was Used to Compute *P* Values

Data	Groups		*P*
	Control (degrees)	Fractured (degrees)	
AP distance between anchoring points (mm)	117 (80-237)	167 (130-217)	0.0061*
AP upper contouring	0 (0-4)	0 (0-22)	0.0407*
AP lower contouring	0 (0-41)	0 (0-15)	0.5144
Lateral distance between anchoring points (mm)	122.5 (86-262)	171 (128-210)	0.0074*
Lateral upper contouring	0 (0-43)	10 (0-27)	0.1526
Lateral lower contouring	0 (0-26)	4 (0-13)	0.0306*
Preoperative Cobb angle	81.5 (23-120)	65 (33-110)	0.7574
Postoperative Cobb angle	51 (4-72)	55 (22-65)	0.6955

* Statistically significant *P* values.

AP indicates anteroposterior.

## DISCUSSION

This is the first study to analyze the mechanisms of rod fracture failure and the risk factors for this in MCGRs. All fractures occurred due to fatigue failure in correspondence with a mechanical indentation, in lengthy and highly contoured implants.

MCGRs can be implanted in single or double-rod configurations. Lately, a double-rod configuration has been preferred over the single one due to the known increased risk of complications associated with the use of single-rod constructs.^15^–^17^ In some patients, in contrast, insertion of 2 constructs may not be achievable due to very low body mass index patients without sufficient soft tissue surrounding the constructs or curve characteristics, which prevent rod insertion. At our implant centre, 8 fractured implants were retrieved, of which 7 were single constructs. Dual rod implants were excluded from the comparative analysis due to the sample size of one. The dual rod construct retrieved, differently from the single ones, fractured on the proximal side of the implant, due to bending fatigue failure. It is important to highlight the fact that most constructs retrieved at our centre were double rod constructs. Single-rod configuration represents a risk factor for rod fracture implant failure.

MCGRs are usually explanted due to an implant failure, infection, or after the end of treatment. Implant failure may occur for several reasons such as actuator pin fracture,^18^,^19^ internal wear and damage,^12^ rods pullout from the fixation devices, and rod fracture. The complication profile and failure rate for these implants still remain unclear and further studies analyzing the overall reasons for failure are needed, combining retrieval, clinical, and imaging data.

Few previous clinical studies have investigated rod fracture in MCGRs. One study focused on the correlation between rod diameter and fracture rate.^20^ Accordingly, with our results, it concluded there is no clear association between rod fracture and rod diameter/size. It also analyzed other clinical and implant parameters and found no correlation between body mass index, lengthenings, and rod fracture rate. Interestingly, the only risk factor associated with rod fracture in that study was conversion status, also known as rod exchange, which seems to be a risk factor in our study as well. Engineering theory would suggest rod diameter to be a greater risk factor for rod fracture, yet in this study, we did not observe a greater incidence of rod fractures in rods with a thinner diameter. Other factors, such as implantation configuration and technique, seem to play a greater role in rod fracture failure mechanisms.

Our study concluded all MCGRs underwent rod fracture failure due to fatigue failure. This is the first study to investigate the fracture failure mode in MCGRs, extensively investigated in other growing rod constructs.^8^,^21^,^22^ Crack initiation points and propagation waves were evident in all the retrieved constructs. The crack initiation point always corresponded to a mechanical indentation most probably induced by the bending tool during the rod contouring procedure; this may necessitate a need to modify the tool or rod bending processes.

Finally, the degree of rod contouring and the distance between anchoring points were computed and a comparison between the control and the fractured groups was carried on. The lateral surface rod indentation and the use of a single rod construct could not fully describe the rod fracture mechanism of failure. All MCGRs fractured in correspondence with the most caudad area of the implant, just above the anchoring point, where supposedly the bending moment is higher due to increased lever arm. Rod contouring and the distance between the anchoring points, where the weight forces are exerted, give a rough estimation of the lever arm, to which the rod is subjected. Our study confirms that the fractured rods had a significantly higher rod contouring and distance between anchoring points compared with the control group, suggesting that it is preferable to implant double rod constructs in patients with sufficient spinal maturity to get lengthy implants.

Limitations of this study include the relatively small sample size and the lack of sufficient retrieved fractured double rod constructs to perform a comparison between the 2 different implant configurations.

This is the first study to investigate the mechanisms of failure of explanted, fractured MCGRs and to assess eventual risk factors. We found fatigue failure as the failure mode for all fractured implants, with the crack initiation point in correspondence with mechanical indentation marks on the rod, probably induced during implantation surgery. We also found that fractured constructs, usually revisions of previous implants, had a significantly greater distance between anchoring points and more pronounced contouring compared with the control group. These results suggest the use of double rod constructs when proceeding with rod exchange if the patient’s characteristics allow it.

## References

[1] Jenks M Craig J Higgins J . The MAGEC system for spinal lengthening in children with scoliosis: a NICE medical technology guidance. Appl Health Econ Health Policy. 2014;12:587–599. 25172432 10.1007/s40258-014-0127-4 PMC4232741

[2] Yang S Andras LM Redding GJ . Early-onset scoliosis: a review of history, current treatment, and future directions. Pediatrics. 2016;137:1–12 10.1542/peds.2015-0709 26644484

[3] Tognini M Hothi H Dal E . Understanding the implant performance of magnetically controlled growing spine rods : a review article. Eur Spine J. 2021;30:1799–1892 33666742 10.1007/s00586-021-06774-8

[4] Zhang YB Zhang JG . Treatment of early-onset scoliosis: techniques, indications, and complications. Chin Med J (Engl). 2020;133:351–357. 31904727 10.1097/CM9.0000000000000614 PMC7004623

[5] Yang JS Sponseller PD Thompson GH . Growing rod fractures: risk factors and opportunities for prevention. Spine. 2011;36:1639–1644. 21738096 10.1097/BRS.0b013e31822a982f

[6] Hill G Nagaraja S Akbarnia BA . Retrieval and clinical analysis of distraction-based dual growing rod constructs for early-onset scoliosis. Spine J. 2017;17:1506–1518. 28456673 10.1016/j.spinee.2017.04.020

[7] Bess S Akbarnia BA Thompson GH . Complications of growing-rod treatment for early-onset scoliosis: analysis of one hundred and forty patients. J Bone Joint Surg A. 2010;92:2533–2543. 10.2106/JBJS.I.01471 20889912

[8] Hosseini P Pawelek JB Nguyen S . Rod fracture and lengthening intervals in traditional growing rods: is there a relationship? Eur Spine J. 2017;26:1690–1695. 27761645 10.1007/s00586-016-4786-8

[9] Agarwal A Jayaswal A Goel VK . Patient-specific distraction regimen to avoid growth-rod failure. Spine. 2018;43:E221–E226. 28614278 10.1097/BRS.0000000000002286

[10] Hosseini P Akbarnia BA Nguyen S . Construct levels to anchored levels ratio and rod diameter are associated with implant-related complications in traditional growing rods. Spine Deform. 2018;6:320–326. 29735144 10.1016/j.jspd.2017.11.004

[11] Hill G Nagaraja S Bridges A . Mechanical performance of traditional distraction-based dual growing rod constructs. Spine J. 2019;19:744–754. 30219359 10.1016/j.spinee.2018.09.006

[12] Wei JZ Hothi HS Morganti H . Mechanical wear analysis helps understand a mechanism of failure in retrieved magnetically controlled growing rods: a retrieval study. BMC Musculoskelet Disord. 2020;21:1–11. 10.1186/s12891-020-03543-4 PMC7409688 32758204

[13] Thakar C Kieser DC Mardare M . Systematic review of the complications associated with magnetically controlled growing rods for the treatment of early onset scoliosis. Eur Spine J. 2018;27:2062–2071. 29675673 10.1007/s00586-018-5590-4

[14] Roye BD Marciano G Matsumoto H . Is rod diameter associated with the rate of rod fracture in patients treated with magnetically controlled growing rods? Spine Deform. 2020;8:1375–1384. 32562099 10.1007/s43390-020-00161-x

[15] Subramanian T Ahmad A Mardare DM . A six-year observational study of 31 children with early-onset scoliosis treated using magnetically controlled growing rods with a minimum follow-up of two years. Bone Jt J. 2018;100B:1187–1200. 10.1302/0301-620X.100B9.BJJ-2018-0031.R2 30168755

[16] Teoh KH Winson DMG James SH . Magnetic controlled growing rods for early-onset scoliosis: a 4-year follow-up. Spine J. 2016;16:S34–S39. 26844638 10.1016/j.spinee.2015.12.098

[17] Cheung JPY Cheung KMC . Current status of the magnetically controlled growing rod in treatment of early-onset scoliosis: what we know after a decade of experience. J Orthop Surg. 2019;27:1–10. 10.1177/2309499019886945 31797729

[18] Jones CS Stokes OM Patel SB . Actuator pin fracture in magnetically controlled growing rods: two cases. Spine J. 2016;16:e287–e291. 26707076 10.1016/j.spinee.2015.12.020

[19] Panagiotopoulou VC Tucker SK Whittaker RK . Analysing a mechanism of failure in retrieved magnetically controlled spinal rods. Eur Spine J. 2017;26:1699–1710. 28102447 10.1007/s00586-016-4936-z

[20] Roye BD Marciano G Matsumoto H . Is rod diameter associated with the rate of rod fracture in patients treated with magnetically controlled growing rods? Spine Deform. 2020;8:1375–1384. 32562099 10.1007/s43390-020-00161-x

[21] Yamanaka K Mori M Yamazaki K . Analysis of the fracture mechanism of Ti-6Al-4V alloy rods that failed clinically after spinal instrumentation surgery. Spine. 2015;40:E767–E773. 25785960 10.1097/BRS.0000000000000881

[22] Shinohara K Takigawa T Tanaka M . Implant failure of titanium versus cobalt-chromium growing rods in early-onset scoliosis. Spine. 2016;41:502–507. 26966974 10.1097/BRS.0000000000001267

